# Surgical Antibiotic Prophylaxis: A Proposal for a Global Evidence-Based Bundle

**DOI:** 10.3390/antibiotics13010100

**Published:** 2024-01-19

**Authors:** Massimo Sartelli, Federico Coccolini, Francesco M. Labricciosa, AbdelKarim. H. Al Omari, Lovenish Bains, Oussama Baraket, Marco Catarci, Yunfeng Cui, Alberto R. Ferreres, George Gkiokas, Carlos Augusto Gomes, Adrien M. Hodonou, Arda Isik, Andrey Litvin, Varut Lohsiriwat, Vihar Kotecha, Vladimir Khokha, Igor A. Kryvoruchko, Gustavo M. Machain, Donal B. O’Connor, Iyiade Olaoye, Jamal A. K. Al-Omari, Alessandro Pasculli, Patrizio Petrone, Jennifer Rickard, Ibrahima Sall, Robert G. Sawyer, Orlando Téllez-Almenares, Fausto Catena, Walter Siquini

**Affiliations:** 1 Department of Surgery, Macerata Hospital, 62100 Macerata, Italy; walter.siquini@sanita.marche.it; 2 General, Emergency and Trauma Surgery Unit, Pisa University Hospital, 56124 Pisa, Italy; federico.coccolini@unipi.it; 3 Global Alliance for Infections in Surgery, 62100 Macerata, Italy; labricciosafrancesco@gmail.com; 4 Department of General Surgery, Faculty of Medicine, Jordan University of Science and Technology, Irbid 22110, Jordan; akomari@just.edu.jo; 5 Department of General Surgery, Maulana Azad Medical College, New Delhi 110002, India; lovenishbains@gmail.com; 6 Department of General Surgery, Bizerte Hospital, Bizerte 7000, Tunisia; oubaraket@gmail.com; 7 General Surgery Unit, Sandro Pertini Hospital, 00157 Rome, Italy; marco.catarci@aslroma2.it; 8 Department of Surgery, Tianjin Nankai Hospital, Nankai Clinical School of Medicine, Tianjin Medical University, Tianjin 300052, China; yunfengcuidoctor@aliyun.com; 9 Department of Surgery, University of Buenos Aires, Buenos Aires 1428, Argentina; aferreres@hospitaldeclinicas.uba.ar; 10 Department of Surgery, Medical School, “Aretaieio” Hospital, National and Kapodistrian University of Athens, 11528 Athens, Greece; georgiokas@yahoo.com; 11 Department of Surgery, Faculdade de Ciências Médicas e da Saúde de Juiz de Fora, Hospital Universitário Terezinha de Jesus, Juiz de Fora 25520, Brazil; caxiaogomes@gmail.com; 12 Department of Surgery, Faculty of Medicine, University of Parakou, Parakou 03 BP 10, Benin; hodasm98@gmail.com; 13 Department of Surgery, Istanbul Medeniyet University, Istanbul 34000, Turkey; arda.isik@medeniyet.edu.tr; 14 Department of Surgical Diseases No. 3, Gomel State Medical University, 246000 Gomel, Belarus; aalitvin@gmail.com; 15 Department of Surgery, Faculty of Medicine, Siriraj Hospital, Mahidol University, Bangkok 10700, Thailand; varut.loh@mahidol.ac.th; 16 Department of General Surgery, Catholic University of Health and Allied Sciences, Mwanza P.O. Box 1464, Tanzania; viharkotecha@gmail.com; 17 General Surgery Unit, Podhalanski Specialized Hospital, 34-400 Nowy Targ, Poland; vladimirkhokha@gmail.com; 18 Department of Surgery No. 2, Kharkiv National Medical University, 61000 Kharkiv, Ukraine; ikryvoruchko60@gmail.com; 19 Department of Surgery, Universidad Nacional de Asuncion, San Lorenzo 1055, Paraguay; gmmachain@yahoo.com; 20 Department of Surgery, School of Medicine, Trinity College, D02 PN40 Dublin, Ireland; oconnd15@tcd.ie; 21 Department of Surgery, University of Ilorin Teaching Hospital, Ilorin 240101, Nigeria; tunde_olaoye_dr@yahoo.com; 22 Medical College, Al-Balqa Applied University, Al-Hussein Hospital, Zarqa 13313, Jordan; jamal.omari@bau.edu.jo; 23 Department of Precision and Regenerative Medicine and Ionian Area (DiMePre-J), Unit of Academic General Surgery “V. Bonomo”, University of Bari “A. Moro”, 70125 Bari, Italy; alessandro.pasculli@policlinico.ba.it; 24 Department of Surgery, NYU Grossman Long Island School of Medicine, NYU Langone Hospital—Long Island, Mineola, NY 11501, USA; patrizio.petrone@nyulangone.org; 25 Department of Surgery, University of Minnesota, Minneapolis, MN 55455, USA; gehr0059@umn.edu; 26 Department of General Surgery, Military Teaching Hospital, Dakar 3006, Senegal; sall_i17@yahoo.fr; 27 Department of Surgery, School of Medicine, Western Michigan University, Kalamazoo, MI 49008, USA; robert.sawyer@wmed.edu; 28 General Surgery Department of Saturnino Lora Provincial Hospital, University of Medical Sciences of Santiago de Cuba, 26P2+J7X, Santiago de Cuba 90100, Cuba; orlandotellez.al@gmail.com; 29 Department of Surgery, “Bufalini” Hospital, 47521 Cesena, Italy; faustocatena@gmail.com

**Keywords:** healthcare-associated infections, surgical site infections, surgical antibiotic prophylaxis, bundle, prevention

## Abstract

In the multimodal strategy context, to implement healthcare-associated infection prevention, bundles are one of the most commonly used methods to adapt guidelines in the local context and transfer best practices into routine clinical care. One of the most important measures to prevent surgical site infections is surgical antibiotic prophylaxis (SAP). This narrative review aims to present a bundle for the correct SAP administration and evaluate the evidence supporting it. Surgical site infection (SSI) prevention guidelines published by the WHO, CDC, NICE, and SHEA/IDSA/APIC/AHA, and the clinical practice guidelines for SAP by ASHP/IDSA/SIS/SHEA, were reviewed. Subsequently, comprehensive searches were also conducted using the PubMed^®^/MEDLINE and Google Scholar databases, in order to identify further supporting evidence-based documentation. The bundle includes five different measures that may affect proper SAP administration. The measures included may be easily implemented in all hospitals worldwide and are based on minimal drug pharmacokinetics and pharmacodynamics knowledge, which all surgeons should know. Antibiotics for SAP should be prescribed for surgical procedures at high risk for SSIs, such as clean–contaminated and contaminated surgical procedures or for clean surgical procedures where SSIs, even if unlikely, may have devastating consequences, such as in procedures with prosthetic implants. SAP should generally be administered within 60 min before the surgical incision for most antibiotics (including cefazolin). SAP redosing is indicated for surgical procedures exceeding two antibiotic half-lives or for procedures significantly associated with blood loss. In principle, SAP should be discontinued after the surgical procedure. Hospital-based antimicrobial stewardship programmes can optimise the treatment of infections and reduce adverse events associated with antibiotics. In the context of a collaborative and interdisciplinary approach, it is essential to encourage an institutional safety culture in which surgeons are persuaded, rather than compelled, to respect antibiotic prescribing practices. In that context, the proposed bundle contains a set of evidence-based interventions for SAP administration. It is easy to apply, promotes collaboration, and includes measures that can be adequately followed and evaluated in all hospitals worldwide.

## 1. Introduction

Healthcare-associated infections (HAIs) have a meaningful impact on health systems, posing a public health threat worldwide [1]. Surgical site infections (SSIs), central-line-associated bloodstream infections, catheter-associated urinary tract infections, ventilator-associated pneumonia, hospital-acquired pneumonia, and *Clostridioides difficile* infections (CDIs) account for most HAIs [2]. Some HAIs are preventable; therefore, these infections can be considered a critical quality patient-care indicator. In 2018, Schreiber et al. [3] published a meta-analysis evaluating the impact of multimodal interventions on reducing HAIs in acute or chronic care settings. They demonstrated a potential HAI rate reduction, ranging from 35% to 55%, when implementing multimodal interventions, notwithstanding the country income level. Regarding SSIs, thirty-six before-and-after studies and one randomised control trial were included in the meta-analysis. The data demonstrated a significant reduction in SSI rates in all countries independently from their economic income group, but differences between subgroups could not be explored due to high heterogeneity. The four studies reporting aggregated SSI rates demonstrated a reduction in SSI rates ranging from 31% to 84% [3]. Although additional higher-quality evidence is required to drive infection prevention efforts from a governance perspective, the results of that meta-analysis should motivate hospitals to implement infection prevention by developing their own multifaceted strategies. 

SSIs represent the most common HAIs occurring in surgical patients [4]. However, while SSI rates seem to be declining in high-income countries, this reduction is not reflected in low- and middle-income countries (LMICs) [5]. SSI rates in LMICs range from 8% to 30% [6]. In 2018, a prospective, international, multicentre cohort study about SSIs after gastrointestinal surgery in high-, middle-, and low-income countries was published. The incidence of SSIs varied significantly between countries with high, middle, and low rankings on the UN’s Human Development Index [5]. Following risk factor adjustment, patients in low-income countries were those at higher risk of SSIs [5]. SSIs may have substantial morbidity, mortality, and economic impacts in these settings.

SSI prevention measures should be integrated before, during, and after surgery. 

Both the World Health Organization (WHO) [7,8,9] and the Centers for Disease Control and Prevention (CDC) [10] have published guidelines for SSI prevention. In 2016, the American College of Surgeons and the Surgical Infection Society updated their SSI guidelines [11]. In 2019, the National Institute for Health and Care Excellence (NICE) published its new guidelines for SSI management online [12]. In 2023, a new set of joint guidelines for SSI prevention in acute-care environments was jointly published [13] by the Society for Healthcare Epidemiology of America (SHEA), the Infectious Diseases Society of America (IDSA), the Association for Professionals in Infection Control and Epidemiology (APIC), and the American Hospital Association (AHA). The evidence-based recommendations stated in these guidelines should be adopted by all healthcare providers caring for patients across the surgical pathway throughout all stages of patient surgical care. 

Surgical antibiotic prophylaxis (SAP) is one of the most important measures to prevent SSIs. SAP consists of administering an antibiotic in patients without active infections before the intervention. Antibiotics for SAP have no therapeutic purposes but are only preventive, aiming to reduce the surgical field microbial burden so that the host defences are not overcome. Ideally, an antibiotic for SAP should be able to [14] achieve the following:

- Prevent SSIs;
- Reduce SSI morbidity and mortality;
- Diminish healthcare duration and cost;
- Not produce any adverse effects;
- Have no aftermath for the patient’s intestinal microbial flora or the healthcare facility.

To achieve these goals, an antibiotic administered for SAP should fulfil the following:

- Active against the most likely bacteria that can contaminate the surgical field;
- Provided in an appropriate dosage and time that ensures adequate serum and tissue concentrations amid the whole operation;
- Safe;
- Administered for the shortest effective period, minimising adverse effects, opportunistic infections, antimicrobial resistance (AMR) development, and costs.

In their clinical practice, surgeons are responsible for many processes of healthcare impacting the risk of SSIs and play a key role in their prevention. However, many surgeons believe that SAP is peripheral to their clinical practice. In fact, using antibiotics properly is essential because their inappropriate use can cause serious side effects and predispose patients to opportunistic infections such as CDI and AMR development and spread.

The microbiome’s indigenous bacteria have a vital host defence role because they can inhibit colonisation by potentially pathogenic bacteria. Nevertheless, opportunists can compromise the microbiota in certain circumstances, meaning it no longer protects against colonisation. Antibiotics can produce a heavy selection pressure on the human microbiome, predisposing patients to AMR, and have considerable consequences for the gut microbiota. While susceptible bacteria can be destroyed, antibiotic pressure can promote pathogenic bacterial overgrowth that may be multidrug-resistant. Moreover, antibiotics can facilitate resistance gene transmission, conferring resistance to other bacteria [14].

SAP is not necessary for all surgical procedures and must be tendered according to well-defined principles. The over-administration of SAP frequently occurs worldwide and contributes to overall antibiotic consumption in surgical units [14]. Given that approximately 15% of all antibiotics prescribed in hospital settings are allocated to SAP, it can be a crucial driver of AMR in these environments [15]. A comprehensive clinical practice guideline for SAP was published in 2015 by the American Society of Health-System Pharmacists (ASHP), the Infectious Diseases Society of America (IDSA), the Surgical Infection Society (SIS), and the Society for Healthcare Epidemiology of America (SHEA) [16]. However, elevated SAP prescribing practice rates that are not compliant with guidelines are common in surgical units globally [17,18,19,20,21,22].

A quality improvement study published in 2019, analysing 9351 surgical episodes and 15,395 prescriptions, found high rates of inappropriate procedural and post-procedural antibiotic use across various Australian hospitals, patients, and surgical factors. The most common reasons for inappropriate SAP were incorrect timing (44.9%), incorrect dosing (26.1%), or an antibiotic spectrum that was too broad (15.9%). Only 65.6% of surgical episodes included a documented incision time [23].

Notably, an ethical mandate to comply with proper and adequate SAP should be considered, representing good clinical practice and correct behaviour. This ethical mandate should be grounded in ethical principles, as collated by Beauchamp and Childress [24]. Here, beneficence stands for “doing the good”, non-maleficence is represented by the “*Primum non nocere*” (“Do no harm”) dictum, and justice means the search for a greater good and the adequate distribution of resources. 

In the multimodal strategy, to implement HAI prevention, bundles are among the most commonly used methods [25] to adapt guidelines in the local context and transfer best practices into routine clinical care. The bundle concept was developed in 2001 by the Institute for Healthcare Improvement (IHI) to support healthcare professionals in improving patient care during specific high-risk treatments. As a general principle, a care bundle should include a set of evidence-based measures that, when implemented together, can produce better outcomes and have a more meaningful impact than the implementation of isolated individual actions [25]. It should be easy to apply, simple, clear, concise, and promote multidisciplinary collaboration. It should be implemented collectively according to an “all or none” approach to accomplish the most favourable outcome and include measures appropriate to the local setting that can be adequately followed and evaluated, with compliance to the bundle assessed by healthcare workers involved in the team. Bundles used as standalone interventions or as part of multimodal strategies are associated with decreased SSI rates [26,27,28]. 

## 2. Methods

This narrative review proposes a bundle with evidence-based measures for SAP that is easily applicable and helpful to improve antibiotic prescribing practices among surgeons from around the world. 

The best strategies for antimicrobial stewardship are not definitively established, and can vary based on local culture, routine clinical practice, and hospital resources. Therefore, it is essential to involve experts worldwide in compiling a document including measures applicable for surgeons in all regions of the world.

An international working group of 30 physicians was established by the Global Alliance for Infections in Surgery in order to define a global evidence-based bundle for appropriate SAP administration. This bundle includes five different actions that may affect adequate SAP administration. The reported measures are based on minimal knowledge of pharmacokinetics and pharmacodynamics, which should be held by all physicians regardless of discipline.

SSI prevention guidelines published by the WHO [7,8,9], CDC [10], NICE [12], and SHEA/IDSA/APIC/AHA [13], and the clinical practice guidelines for SAP by ASHP/IDSA/SIS/SHEA [16], were reviewed. Subsequently, comprehensive searches were also conducted using the PubMed^®^/MEDLINE (National Library of Medicine, Bethesda, MD, USA) and Google Scholar (Alphabet, Inc., Mountain View, CA, USA) databases, in order to identify further supporting evidence-based documentation. The search term used was “surgical antibiotic prophylaxis”. Overall, 5670 articles published in the English language between January 2012 and November 2023 were identified. Two authors selected 462 abstracts. In addition to the above-mentioned SSI prevention guidelines, 71 articles were reviewed to prepare the first draft. The resulting document was shared with all the members of the working group, thoroughly reviewed, and finally approved. 

## 3. A Proposal for a Global Evidence-Based Bundle

**Figure 1:**
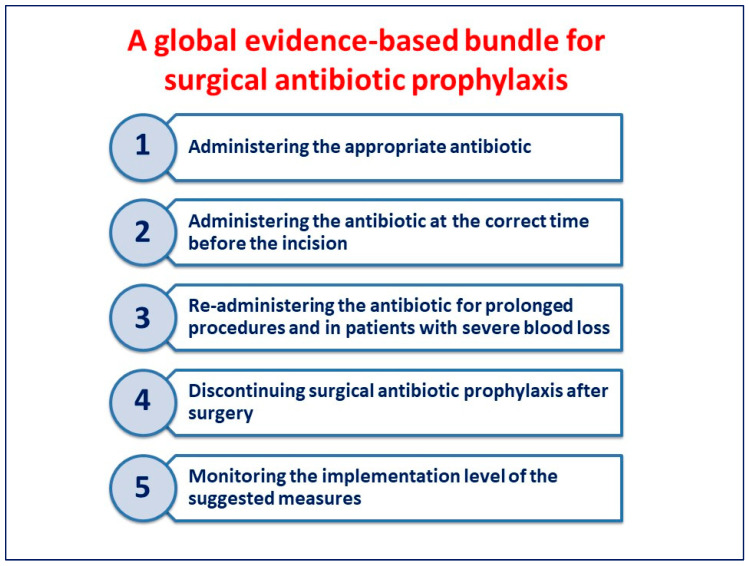
A global evidence-based bundle for surgical antibiotic prophylaxis.

The measures included in the bundle (Figure 1) may be easily implemented in all hospitals worldwide.

### 3.1. Administering the Appropriate Antibiotic

The risk of SSIs [29] may differ depending on the site and degree of colonisation or contamination of the surgical procedure. Surgical procedures can be divided into four classes, categorised as clean (Class I), clean/contaminated (Class II), contaminated (Class III), and dirty (Class IV) [29].

SAP should be prescribed for surgical procedures at high risk OF SSIs, such as clean–contaminated and contaminated surgical procedures or for clean surgical procedures where SSIs, even if unlikely, may have devastating consequences, such as in procedures with prosthetic implants. SAP should also be prescribed in patients with medical conditions associated with a higher risk of SSI, such as immunocompromised patients [29]. 

The route of SAP administration may vary with the type of procedure. However, intravenous administration is ideal for most procedures because it produces rapid and predictable antibiotic tissue concentrations [16]. SAP in patients undergoing open-groin hernia surgery has been debated with conflicting results of low evidence quality [30,31,32,33,34,35]. The 2018 HerniaSurge Group International guidelines for groin hernia management recommended SAP in open-groin mesh repair in any patient in a high-risk infection environment [36]. A Cochrane systematic review of SAP for preventing SSIs in adults undergoing open elective inguinal or femoral hernia repair was published in 2020 [33]. The systematic review investigated three outcomes: superficial SSIs, deep SSIs, and all SSIs (superficial SSIs + deep SSIs). Very low-quality evidence demonstrated that it is uncertain whether SAP reduces the risk of all SSIs after hernia surgery. Moderate-quality evidence demonstrated that SAP makes little difference in reducing the risk of all SSIs after hernia surgery in a low-risk infection environment. Low-quality evidence showed that SAP in a high-risk environment may reduce the risk of all SSIs and superficial SSIs. Very low-quality evidence demonstrated that it is uncertain whether SAP can reduce deep SSIs after hernia surgery [29]. In sum, SAP should be performed in patients undergoing hernia surgery in a high-risk infection environment, but not in patients undergoing hernia surgery in a low-risk infection environment.

Another topic debated with conflicting results has been whether to prescribe SAP in patients undergoing laparoscopic cholecystectomy. Current evidence does not recommend the routine prescription of SAP for elective laparoscopic cholecystectomy for uncomplicated gallstone disease [37,38,39,40], but compliance with this evidence is generally low [41].

Antibiotics prescribed for SAP should be nontoxic, inexpensive, and have in vivo activity against the common bacteria causing SSIs. They should be effective against the most likely bacteria contaminating the surgical field. SSIs following clean interventions are usually due to Gram-positive bacteria commensal skin flora, including Staphylococcus aureus or Streptococcus species [29]. Clean–contaminated and contaminated interventions may be contaminated by various commensal flora bacteria of incised mucosae, such as Escherichia coli or other Enterobacterales and anaerobes bacteria [16]. The WHO [7,8,9] guidelines recommend administering SAP before the surgical incision when it is indicated. The CDC guidelines [10] recommend administering SAP only based on published clinical practice guidelines and timed in such a way as to achieve a bactericidal concentration of antibiotics in the serum and tissues when the incision is made. The SHEA guidelines [13] recommend prescribing appropriate antibiotics for SAP based on surgical procedures, the most common bacteria causing SSIs for a specific operation, and published guidelines. The NICE guidelines [12] recommend not using SAP routinely for clean, non-prosthetic, uncomplicated surgery.

The most commonly used antibiotics for SAP are first- and second-generation cephalosporins, including cefazolin, cefuroxime, cefoxitin, or the combination of cefazolin plus metronidazole, when it is necessary to cover anaerobes such as in colorectal surgery. For most surgical procedures, cefazolin is the antibiotic of choice for SAP. It has the most widely proven efficacy of a studied antibiotic. It is considered by the WHO an essential drug and as such it should be available in every hospital of the world [42]. 

There are few data describing the rate and quality indices of antibiotics used in hospitalised patients in LMICs especially in Africa. However, the few data show that the prevalence of antibiotic use in hospital settings in Africa is higher than the prevalence reported in hospital settings in the other continents [43]. Broad-spectrum antibiotics such as ceftriaxone and fluoroquinolones are antibiotics commonly prescribed in hospitalised patients in Africa [43]. SAP is the second most common indication for antibiotic use in African hospital settings. Therefore, SAP represents an important priority for the implementation of antimicrobial stewardship programmes (ASPs) in this continent [43]. 

A recent prospective trial compared piperacillin–tazobactam with cefoxitin as SAP for pancreatoduodenectomy. Among 778 patients enrolled in the study (378 in the piperacillin–tazobactam group and 400 in the cefoxitin group), the SSI rate at 30 days was lower in the piperacillin–tazobactam group compared with the cefoxitin group [44]. It is important to stress that the use of an antibiotic with such a broad spectrum may be justified for SAP only in complex operations with a very high rate of complications.

Routine use of antifungal agents should be discouraged except for very special circumstances, such as liver transplantation [45]. The routine use of glycopeptides, such as vancomycin or teicoplanin for SAP, should be discouraged. Glycopeptides can be considered for patients known to be colonised by methicillin-resistant *Staphylococcus aureus* (MRSA) or who are likely to have had recent MRSA exposure [29]. Moreover, vancomycin is less effective than cefazolin in preventing SSIs caused by *methicillin-susceptible Staphylococcus aureus* [16].

Establishing which antibiotics to use for patients known to be colonised or to have had past infection with multidrug-resistant (MDR) bacteria is complex and cannot be defined uniformly. Defining if SAP should be prescribed to provide coverage against MDR bacteria depends on many factors, such as bacteria antibiotic susceptibility, the host, and the surgical procedure. While it may be logical to prescribe SAP with an agent active against MRSA for any patient known to be colonised with MRSA who will undergo a skin incision, specific prophylaxis for resistant Gram-negative bacteria in a patient known to be colonised with such bacteria may not be necessary for a purely cutaneous procedure. Thus, patients known to be colonised or to have had past infection with MDR bacteria must be treated on a case-by-case basis, taking into account multiple considerations. Future well-designed clinical studies will assess the SAP effectiveness in patients colonised with MDR bacteria [46]. 

Regarding obese patients, the CDC guidelines [10] do not identify randomised controlled trials that evaluated the benefits of weight-adjusted SAP dosing and its effect on the risk of SSIs. The SHEA guidelines suggest adjusting dosing based on patient weight [13]. Regarding cefazolin, the SHEA guidelines recommend using 2 g dosing for patients weighing ≤ 120 kg and 3 g dosing for patients weighing > 120 kg. Data about the role of 3 g of cefazolin dosing in reducing SSIs in obese patients are conflicting. However, some (low-level) studies have shown a benefit of 3 g dosing compared to 2 g dosing in this patient population, with few adverse events [13]. On the contrary, according to other evidence, in these patients, the choice of the first dose in obese patients should be guided by the pharmacokinetics (especially tissue penetration and volume of distribution) of the individual antibiotics, depending on whether the antibiotic is hydrophilic or lipophilic. Because cefazolin is hydrophilic, penetration into tissue is not dose-dependent. Therefore, high cefazolin doses may not be necessary in obese patients [47,48,49]. In contrast, cefoxitin is not as hydrophilic as cefazolin and higher doses of cefoxitin may be required for obese patients. 

Few data have been published regarding the SAP prescription in patients undergoing solid organ transplantation (SOT) [50,51]. SOT patients are at high risk of early postoperative infections because of the complexity of surgical procedures and therapeutic immunosuppression. SOT patients are also at increased risk of infections caused by MDR bacteria. These risks may lead to liberalised SAP in SOT patients. Perceived overuse of SAP in SOT patients has led to calls for antibiotic stewardship in the organ transplant setting [52].

Beta-lactam antibiotic allergy history should be considered when selecting SAP. Patients should be questioned carefully before the SAP administration about their antibiotic hypersensitivity background to determine whether a true allergy exists. Although up to 10% of patients will report an allergy to penicillin, the incidence of severe adverse reactions is well under 1% [16]. In addition, the patient cross-reactivity between penicillin and cephalosporin or carbapenem hypersensitivity is <5% [16]. The SHEA guidelines [13] recommend obtaining a thorough allergy history because self-reported allergy to beta-lactam antibiotics has been related to a higher risk of SSIs resulting from administering non-beta-lactam agents. Most patients with a self-reported allergy to beta-lactam antibiotics can safely receive a beta-lactam antibiotic as prophylaxis [29]. Non-beta-lactam agent alternatives include clindamycin, gentamicin, vancomycin, or fluoroquinolones. Vancomycin has a broad anti-Gram-positive activity; however, it is less effective than cefazolin at treating methicillin-susceptible *Staphylococcus aureus infections* [29]. Additionally, vancomycin and gentamicin are linked with a risk of antibiotic-associated nephrotoxicity, which has been reported in patients receiving only a few doses of SAP [53]. Clindamycin is the most frequently prescribed antibiotic in patients with a documented beta-lactam allergy. However, clindamycin resistance to *Staphylococcus aureus* is increasing. This can decrease its efficacy against this pathogen often isolated in SSIs [53]. Clindamycin has also been reported to be associated with a nearly three-fold increased risk of CDI compared to other antibiotics [54]. Even single doses of clindamycin used for SAP have been associated with an increased risk of CDI. Consequently, appropriately evaluating allergies to beta-lactam antibiotics to limit unnecessary clindamycin exposure in surgical patients is essential to mitigate the risk of CDI [53].

Topical antibiotic prescription remains common among surgeons despite no evidence of efficacy. A systematic review and meta-analysis on the topical antibiotic prophylaxis use for SSI prevention in clean and clean–contaminated surgery was published in 2022 [54]. Thirteen randomised control trials (RCTs) comparing topical antibiotic agents with non-antibiotic agents were evaluated through the meta-analysis. As per the current evidence, administering topical antibiotic agents to surgical wounds does not diminish SSI incidence. The NICE [12], the CDC [10], and the WHO [7,8,9] guidelines recommend avoiding the use of topical antibiotic agents to prevent SSIs.

Oral antibiotic bowel preparation (oABP) for elective colonic surgery has been debated recently and merits particular consideration. oABP has been prescribed in addition to mechanical bowel preparation (mBP) and intravenous antibiotics [29]. Although the oABP–mBP combination has been employed widely in North America, it has been used less in Europe, perhaps because Enhanced Recovery After Surgery (ERAS^®^) protocols omit routine mBP in patients’ preparation. The WHO guideline panel suggests that the oABP–mBP combination should be used in adult patients undergoing elective colorectal surgery to prevent SSIs. Nonetheless, the guidelines recommend the non-use of mBP alone for SSI prevention in adult patients undergoing elective colorectal surgery [7,8,9]. The SHEA guidelines recommend parenteral and oral combination use before elective colorectal surgery to prevent SSIs [13]. A Cochrane meta-analysis enrolling 21 RCTs with 5264 adult patients undergoing elective colorectal surgery was published in 2022 [55]. The meta-analysis compared mBP plus oABP with either mBP alone, oABP alone, or no bowel preparation. Based on moderate-certainty evidence, the meta-analysis results suggest that mBP plus oABP may be more effective than mBP alone in preventing SSIs. However, the meta-analysis was unable to clarify whether oABP alone is equivalent to MBP + oABP, because of the low to very low quality of evidence. A weighty limitation of oABP standardisation is the heterogeneity of the data about the choice of antibiotics and the duration. Antibiotics, dosages, and timing are very heterogeneous, making the results difficult to summarise. These aspects have yet to be defined by evidence [29].

### 3.2. Administering the Antibiotic at the Correct Time before the Incision

Adequate tissue concentrations of antibiotics should be present at the surgical site throughout the surgical procedure. The WHO global guidelines recommend administering SAP before surgical incision when indicated (depending on the type of operation). These guidelines recommend SAP administration within 120 min before the incision, based on the half-life of the prescribed antibiotic. A meta-analysis published in 2017 evaluated the proper SAP timing and compared the different administration time intervals [56]. Fourteen observational studies, including 54,552 patients, were included in this review (thirteen of these studies were included in the meta-analysis conducted by WHO experts). The study did not show a significant difference when SAP was tendered 120–60 min before surgical incision compared to when SAP was administered 60–0 min before surgical incision. However, the SSI risk doubled when antibiotics were issued after the first incision and was five-fold higher when they were furnished more than 120 min before the incision. 

Weber et al. in 2017 [57] published a randomised controlled trial evaluating the optimal SAP timing consisting of a single 1.5 g dose of cefuroxime (short half-life cephalosporin) given through intravenous infusion associated with 500 mg of metronidazole in colorectal surgery. The trial demonstrated that early antibiotic administration for SAP did not significantly reduce the SSI risk compared with late administration, not supporting any 60 min window in administering a short-half-life cephalosporin for SAP. The SHEA guidelines [13] recommend administering antibiotics within 1 h of incision to optimise the tissue concentration.

The first antibiotic dose should always be administered within 60 min, according to the prescribed antibiotic pharmacokinetics, before surgical incision for most commonly used antibiotics (including cefazolin). This can guarantee appropriate tissue concentrations during the surgical intervention. Only drugs with more extended half-lives, such as vancomycin, should be issued more than 60 min before the incision. The ideal time to administer preoperative cefazolin has been investigated recently in an interesting pharmacological study. According to the study, cefazolin reaches its peak concentration 40 min after intravenous administration, and then immediately decreases, remaining effective for 4 h [58].

### 3.3. Re-Administering the Antibiotic for Prolonged Procedures and in Patients with Severe Blood Loss

The NICE guidelines [12] recommend considering the antibiotic pharmacokinetics in SAP prescription. They also recommend administering a repeat SAP dose when the operation lasts longer than the administered antibiotic half-life. Although, in 2017, the CDC [10] did not identify sufficient high-quality evidence to evaluate the intraoperative redosing benefits of SAP for SSI prevention, from a pharmacokinetic standpoint, additional intraoperative doses should be issued for procedures exceeding two antibiotic half-lives or for procedures with significant associated blood loss (more than 1.5 L). This can guarantee an antibiotic concentration above the minimal inhibitory concentration at the surgical site throughout the procedure.

A meta-analysis including two randomised controlled trials and eight cohort studies [59] confirmed the importance of antibiotic redosing. Even though there was heterogeneity among the antibiotics administered, SAP intraoperative redosing reduced SSI rates compared with a single preoperative dose in any surgery. In a cefazolin case with a half-life of approximately 2 h, an additional intra-operative dose should be repeated after about 4 h. Conversely, cefoxitin has a very short half-life of 60 min, so the subsequent intra-operative dose should be repeated after roughly 2 h.

### 3.4. Discontinuing SAP after Surgery

SAP aims to prevent SSIs and should be administered and maintained at sufficiently high concentrations at the surgical site during the time that the incision is open. Erroneously, some surgeons believe that prolonging SAP after that the surgical incision has been closed can protect the patient from post-operative infections [29].

No evidence supports SAP use after the surgical procedure. Regardless, continuing SAP after surgery is still very common. Global Point Prevalence Survey results, including adult data from 303 hospitals in 53 countries, were published in 2015. This international point prevalence study demonstrated that SAP for more than 24 h ranged from 29.5% in Western Europe to 92.5% in Africa [60]. The WHO global guidelines [7,8,9] recommend not prolonging SAP administration after the operation completion to prevent SSIs. WHO experts conducting a meta-analysis [7,8,9] identified 69 randomised controlled trials researching the optimal antibiotic prophylaxis duration in different surgical procedures to evaluate SSI rate reduction; they found some low- to very low-quality evidence that prolonged postoperative antibiotic administration can be beneficial for reducing SSI risk in cardiac and vascular procedures. Considering the limited evidence, potentially damaging events, or AMR development associated with antibiotic prolongation, the experts advised against postoperative antibiotic administration. The CDC guidelines also recommend not administering additional SAP doses in clean and clean–contaminated procedures after the surgical incision has been closed in the operating room, even in the presence of a drain. Also, the SHEA [13] guidelines recommend stopping antibiotics after the incisional closure in the operating room.

In 2020, a meta-analysis published by de Jonge et al. [61] evaluated the effect of continued SAP on SSI rate. They considered 83 relevant prospective randomised trials, of which 52, with 19,273 participants, were included in the primary meta-analysis. Overall, there was no conclusive evidence identifying a postoperative continuation of SAP having a benefit versus discontinuation when best infection prevention and control practices were followed. A retrospective, single-centre cohort study published in 2021 [62] compared the efficacy of single-dose antibiotic use versus 24 h SAP dosing in patients undergoing total joint arthroplasty. The study’s results displayed no significant differences in patient characteristics between single-dose and 24 h dosing. Between single and 24 h dosing SAP, there were no significant differences in acute periprosthetic joint infection rates, superficial SSI, 90-day reoperation, or 90-day complications. In a multicentre, national, retrospective cohort study published in 2019 [63], increased SAP duration was associated with a higher acute renal failure risk and CDI without reducing SSIs.

### 3.5. Monitoring the Implementation Level of the Suggested Measures

Understanding the infection prevention and control programme effect is essential to ensure it is implemented and executed as designed. Evaluating an action plan impact through surveillance with timely feedback is crucial to infection prevention and control action. This allows hospitals and healthcare professionals to gauge the strategies’ effectiveness.

The appropriateness of prevention measures may depend on healthcare workers’ behaviour and the availability of appropriate environmental and structural organisation. To improve compliance with prevention measures and ensure their long-term sustainability, the frequent assessment of working practices and timely result feedback to stakeholders is crucial. A systematic review of the effective strategies for implementing care bundles was published in 2015. Forty-seven studies were included in the review, and the three most frequently used strategies when a bundle was implemented were education, reminders, and audit and feedback [64]. The SHEA guidelines [13] recommend providing ongoing SSI rate feedback to surgical and perioperative personnel and leadership. Regarding the SSI prevention setting, in 2017, the European Centre for Disease Prevention and Control (ECDC) published [65] an updated version of a technical document (HAI-Net SSI protocol, version 2.2), proposing various process indicators for SSI prevention (including SAP) based on the strength of available evidence and the feasibility of their collection. 

Care bundles are sets of evidence-based recommendations that, when implemented together, can result in better outcomes than when implemented individually. In 2019, a scoping review about barriers and facilitators to successfully implementing care bundles in the hospital setting was published. Bundles with a few simple measures were described to have better compliance rates. Standardising reporting of implementation strategies may help to transfer evidence-based bundle recommendations into clinical practice [66]. To reinforce the need to monitor the implementation level, we have included this concept as the last measure of the bundle. 

ASPs can optimise the treatment of infections and reduce adverse events associated with antibiotics. In the context of a collaborative and interdisciplinary approach, it is essential to encourage an institutional safety culture in which surgeons are persuaded, rather than compelled, to respect antibiotic prescribing practices. 

The proposed bundle contains a set of evidence-based interventions for SAP administration. It is easy to apply, promotes collaboration, and includes measures that can be adequately followed and evaluated in all hospitals worldwide.

## 4. Discussion

Appropriate prescription of antibiotics should be integral to good clinical practice and standards of care. On the contrary, inappropriate antibiotic prescriptions, as well as poor infection prevention and control practices, are contributing to the development and spread of AMR [14].

Evidence has demonstrated that hospital ASPs aimed at improviung antibiotic use can optimise the management of infections and reduce adverse events associated with antibiotic use, including the global burden of AMR [14]. 

Fifteen years after the joint guidelines published by SHEA/IDSA [67], the best strategies for ASPs are still not defined. Moreover, many acute care hospitals worldwide do not have any ASP. The preferred means of improving antimicrobial stewardship should include a comprehensive programme incorporating collaboration among professionals within an institution. In this context, the direct involvement of all prescribers is crucial. Surgical wards represent settings where the use of antibiotics can be optimised. ASPs should include SAP as a critical area for improvement. Standardising a shared antibiotic prophylaxis protocol should be the first step of any ASP. Compliance with this protocol should be audited regularly, and the results should be fed back to the antimicrobial prescribers and decision-makers [68,69].

The systematic review by Davey et al. [70] demonstrated strong evidence that antibiotic use interventions among inpatients were associated with increased antibiotic policy compliance and duration. Of the 159 studies with intervention outcomes, 11 (6.9%) targeted SAP. Interventions were demonstrated to successfully reduce unnecessary antibiotic use in hospitals, even though the majority did not use the most effective behaviour change techniques. Recently, a retrospective study compared the selection and duration of antibiotics for SAP over six months, both before and after a five-year intervention. The rate of appropriate prescription of antibiotics for SAP improved to 80% during the post-intervention period. The rate of correct SAP duration increased significantly, from 69.1% (n = 1598) in the pre-intervention period to 78.0% (n = 841) in the post-intervention period (*p* < 0.001). The prescriptions of third cephalosporins, such as ceftriaxone, significantly decreased, while the prescriptions of cefazolin increased by more than nine times. No increases in SSIs were detected after the intervention. The implementation of an antimicrobial stewardship programme in the surgical ward demonstrated a positive impact on SAP prescriptions [71]. 

Using the best evidence is a fundamental aspect of healthcare quality. Guidelines for clinical practice are essential to disseminate evidence-based practices, improving healthcare quality and safety. Several guidelines have recently been published [5,6,7,8,9,10,11,12,13] regarding preventing SSIs. However, guidelines are not self-implementing, and complying with measures stated in guidelines is often challenging [72]. A systematic review assessing adherence to guidelines for SAP, published in 2015, demonstrated the need for greater adherence to guidelines [73]. A prospective, multicentre cohort study in orthopaedic surgery showed that lack of compliance with SAP guidelines is significantly associated with increased SSI rate [74]. 

Adapting clinical SAP guidelines in the local context may improve acceptance and adherence to best practices, also considering the local microbiological epidemiology. 

The evolving field of implementation research has increasingly addressed how to adapt guidelines to local contexts and translate evidence into practice. Active involvement of the guidelines users in their adaption can lead to significant changes in clinical practice. Adapting clinical guidelines in a local context, such as local protocols or bundles, while specifying responsibilities for particular actions in a hospital setting, may be helpful to engage all professionals in guideline implementation [25]. 

Various implementation interventions have been described and can potentially be used to promote compliance with guidelines [75]. In the setting of SSI prevention, in 2019, Ariyo et al. published a systematic review of utilised implementation strategies [76]. They categorised implementation interventions using the “four Es” approach [74]—“engage”, “educate”, “execute”, and “evaluate”—as the core components of change behaviour. 

In the context of a multimodal strategy to implement HAI prevention, bundles are one of the most commonly used methods to translate guidelines to the local setting. A systematic review of the effect of interventions on the incidence of SSIs in acute care settings was recently published. Twenty-three studies showed that interventions effective in preventing SSIs have multiple components such as care bundles, stakeholder engagement, targeted surveillance, and education [77]. An attractive, comprehensive review of the reasons for poor compliance with guidelines was published by Leaper et al. [78], who reported recommendations to improve patient outcomes and prevent SSIs. These recommendations included the following:

- Tracking compliance with hospital care bundles and conducting qualitative research into reasons for non-compliance with bundles;
- Incorporating checklists and care bundles into the informed consent process to make them as transparent as possible;
- Developing surveillance methods with shared SSI definitions and indicators that can be reliably interpreted in clinical practice and that can promote a benchmarking analysis of anonymised individual surgeon SSI rates;
- Updating national and local guidelines as new evidence evolves;
- Recognising compliant surgery/operating theatre work teams;
- Incorporating checklists and care bundles;
- Planning effective communication strategies with healthcare providers.

In administering antibiotics for any indication, including for SAP, surgeons should always be responsible for handling antibiotics with care.

In this narrative review, an international working group of 30 physicians from many regions of the world has defined an evidence-based bundle for appropriate SAP administration. This bundle includes five actions that may affect adequate SAP administration in all surgical wards worldwide.

1. *Administering the appropriate antibiotic.* SAP should be prescribed for surgical procedures at high risk of SSIs, such as clean–contaminated and contaminated surgical procedures or for clean surgical procedures where SSIs, even if unlikely, may have devastating consequences, such as in procedures with prosthetic implants. SAP should also be prescribed in patients with medical conditions associated with a higher risk of SSI, such as immunocompromised patients. The most commonly used antibiotics for SAP are first- and second-generation cephalosporins, including cefazolin, cefuroxime, cefoxitin, or the combination of cefazolin plus metronidazole, when it is necessary to cover anaerobes such as in colorectal surgery. Patients known to be colonised or to have had past infection with MDR bacteria must be treated on a case-by-case basis, taking into account multiple considerations. Future well-designed clinical studies will assess the SAP effectiveness in patients colonised with MDR bacteria. Although topical antibiotic prescription remains common among surgeons, it should be discouraged.
2. *Administering the antibiotic at the correct time before the incision.* Adequate tissue concentrations of antibiotics should be present at the surgical site throughout the surgical procedure. The first antibiotic dose should always be administered within 60 min before surgical incision for most commonly used antibiotics (including cefazolin). This can guarantee appropriate tissue concentrations during the surgical intervention. Only drugs with more extended half-lives, such as vancomycin, should be issued more than 60 min before the incision.
3. *Re-administering the antibiotic for prolonged procedures and in patients with severe blood loss*. Intraoperative doses should be issued for procedures exceeding two antibiotic half-lives or for procedures associated with blood loss (more than 1.5 L). This can guarantee that the antibiotic concentration is maintained above the minimal inhibitory concentration at the surgical site throughout the procedure.
4. *Discontinuing SAP after surgery*. SAP aims to prevent SSIs and should be administered and maintained at sufficiently high concentrations at the surgical site during the time that the incision is open. Erroneously, some surgeons believe that prolonging SAP after that the surgical incision has been closed can protect the patient from post-operative infections. On the contrary, SAP administration should not be prolonged after the operation completion to prevent SSIs.
5. *Monitoring the implementation level of the suggested measures.* To improve compliance with prevention measures and ensure their long-term sustainability, frequent assessment of working practices and timely result feedback to stakeholders is crucial. As a multimodal strategy to implement HAI prevention, bundles are among the most commonly used methods to adapt guidelines in the local context and transfer best practices into routine clinical care. The proposed bundle contains a set of evidence-based interventions for SAP administration. It is easy to apply, promotes collaboration, and includes measures that can be adequately followed and evaluated in all hospitals worldwide. Major efforts should be made in all hospitals around the world to verify that the proposed measures are implemented in the context of a bundle strategy.

## 5. Conclusions

The use of SAP contributes considerably to the total amount of antibiotics prescribed in hospitals worldwide. Its overuse can be associated with antibiotic-related adverse events, including the development of AMR and elevated healthcare costs. Approximately 15% of all antibiotics in hospitals are prescribed for SAP. Bundles are one of the most commonly used methods to adapt guidelines to the local context and implement SSI prevention.

SAP consists of administering an antibiotic without active infections before the intervention. Antibiotics have no therapeutic purposes but are only preventive, aiming to reduce the surgical field microbial burden so that the host defences are not overcome. 

In this article, we have presented an evidence-based bundle for correct SAP based on a review of the best available evidence. This bundle can be easily applied everywhere and we hope that it can help improve antibiotic prescribing practices among surgeons worldwide.
